# Efficacy of radial artery cannulation in treatment of cardiac arrest: A case report

**DOI:** 10.1097/MD.0000000000039149

**Published:** 2024-08-02

**Authors:** Li Jin, Hanzhen Ji, Jianru Xu

**Affiliations:** a Department of Emergency, Nantong Third People’s Hospital, Affiliated Nantong Hospital 3 of Nantong University, Nantong, Jiangsu, China; b Department of Emergency Medicine, The First Affiliated Hospital of Soochow University, Suzhou, Jiangsu, China; c Department of Library, Nantong Third People’s Hospital, Affiliated Nantong Hospital 3 of Nantong University, Nantong, Jiangsu, China.

**Keywords:** cardiopulmonary resuscitation, invasive arterial pressure monitoring, out-of-hospital cardiac arrest, radial artery cannulation, return of spontaneous circulation

## Abstract

**Rationale:**

Timely treatment and recovery of cardiac arrest in out-of-hospital settings present significant challenges. This report describes a novel method of integrating advanced monitoring techniques such as radial artery cannulation in the treatment of an 85-year-old male patient who suffered an out-of-hospital cardiac arrest (OHCA).

**Patient concerns:**

The patient, an 85-year-old man, experienced sudden cardiac arrest at home around 4:10 pm on November 22, 2023, characterized by immediate loss of consciousness and absence of pulse, and no response when called by name, necessitating urgent medical intervention.

**Diagnoses:**

The patient was diagnosed with OHCA by the emergency doctor, which was further confirmed by the absence of spontaneous circulation and respiratory failure.

**Interventions:**

The patient was treated with manual cardiopulmonary resuscitation (CPR), ventilator-assisted ventilation, internal jugular venous catheterization, medical treatment, mechanical CPR, and supplemented by radial artery cannulation for invasive blood pressure monitoring. This technique was pivotal for real-time hemodynamic assessment.

**Outcomes:**

The invasive monitoring facilitated the early detection of the return of spontaneous circulation, allowing for the timely cessation of mechanical CPR. Subsequent treatment in the intensive care unit was optimized based on continuous arterial pressure readings, enhancing the stabilization of the patient’s condition.

**Lessons:**

This case underscores the significant role of radial artery cannulation for invasive blood pressure monitoring in improving clinical outcomes for patients experiencing OHCA. Integrating radial artery cannulation with other advanced monitoring techniques aids in the early detection of the return of spontaneous circulation and optimizes subsequent intensive care treatment.

## 1. Introduction

Cardiac arrest remains a leading cause of mortality worldwide, and the out-of-hospital cardiac arrest (OHCA) presents a particularly daunting challenge in emergency medical services (EMS) due to its unpredictability and the immediate need for effective resuscitation efforts. The American Heart Association emphasizes the importance of high-quality cardiopulmonary resuscitation (CPR) as a main determinant of both survival and neurological outcome after cardiac arrest.^[1]^ Traditional manual CPR is limited by variability in compression depth and fatigue of the rescuer, thus leading to inconsistent quality as time goes on.

Mechanical CPR devices have been introduced to address these limitations, which can offer the benefit of a consistent and uninterrupted chest compression. The Lund University Cardiac Assist System device, one of the mechanical CPR devices used in clinical practice, has shown promising results by maintaining coronary perfusion pressures and potentially improving cardiac resuscitation outcomes.^[2]^ However, one of the major challenges during the mechanical CPR is the detection of the return of spontaneous circulation (ROSC). Some traditional methods, such as manual pulse checks, can be unreliable due to the mechanical activity produced by the device, which may interfere with the rescuer’s ability to detect a pulse.

The invasive arterial blood pressure monitoring has been proposed as a solution to this challenge, and it offers a real-time assessment of circulatory state in this patient through continuous invasive hemodynamic monitoring, which is particularly valuable during ongoing mechanical chest compression. The use of radial artery cannulation for invasive blood pressure monitoring during CPR has been demonstrated to facilitate the early detection of ROSC, thus potentially leading to more timely and appropriate adjustments in cardiac resuscitation efforts.^[3]^

Despite the potential benefits, the integration of such advanced monitoring techniques during CPR is not yet standard practice in EMS, and the data on its effect on clinical outcomes are limited. This case report aims to describe the successful application of radial artery cannulation for invasive blood pressure monitoring during mechanical CPR in an OHCA patient and further explore its implications for emergency medicine practice. It is intended to contribute to the growing body of evidence supporting the use of mechanical CPR devices and advanced monitoring techniques in the treatment of cardiac arrest.

## 2. Case presentation

An 85-year-old male patient suffered a sudden cardiac arrest at home around 16:10 on November 22, 2023, characterized by immediate loss of consciousness, absence of pulse, cyanosis, and no response when called by name. EMS were contacted at 16:10. Upon their arrival, they immediately diagnosed this patient with cardiac arrest and initiated manual CPR.

Upon admission to the emergency department at 16:35, the patient did not achieve the ROSC. Therefore, the medical team carried out mechanical CPR with The Lund University Cardiac Assist System device and intravenously injected 1 mg adrenaline every 3 minutes (a total dose of 8 mg adrenaline) in this patient. Additionally, this patient was intubated and ventilated.

**Figure 1.**
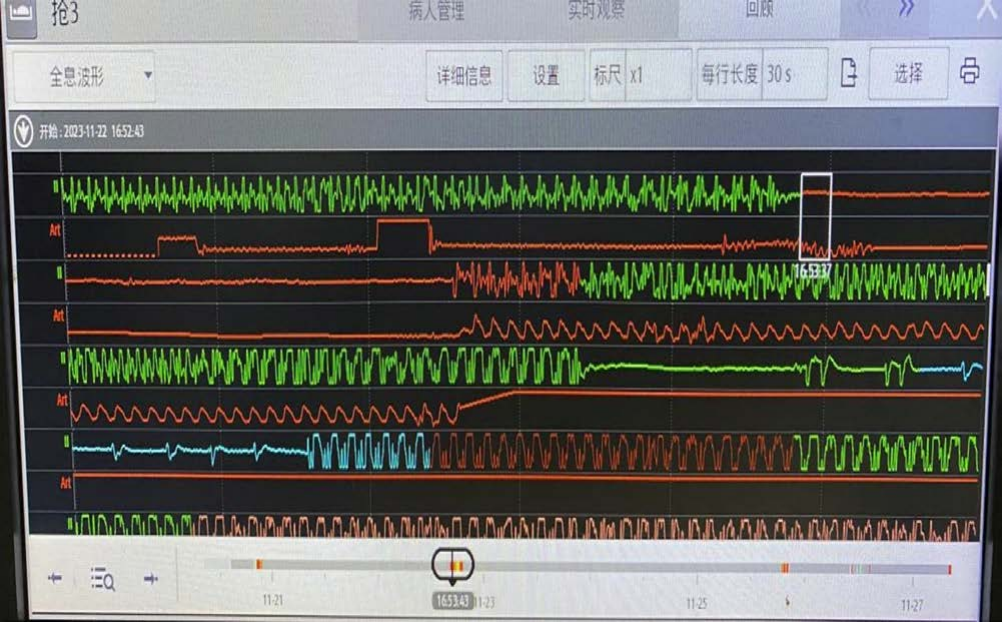
Arterial blood pressure waveform at 16:53 after cannulation with a very small amplitude.

This patient underwent successful radial artery cannulation for invasive blood pressure monitoring at 16:53, which is a critical intervention. The initial arterial pressure waveform observed after radial artery cannulation is of a very small amplitude (see Fig. 1).

**Figure 2.**
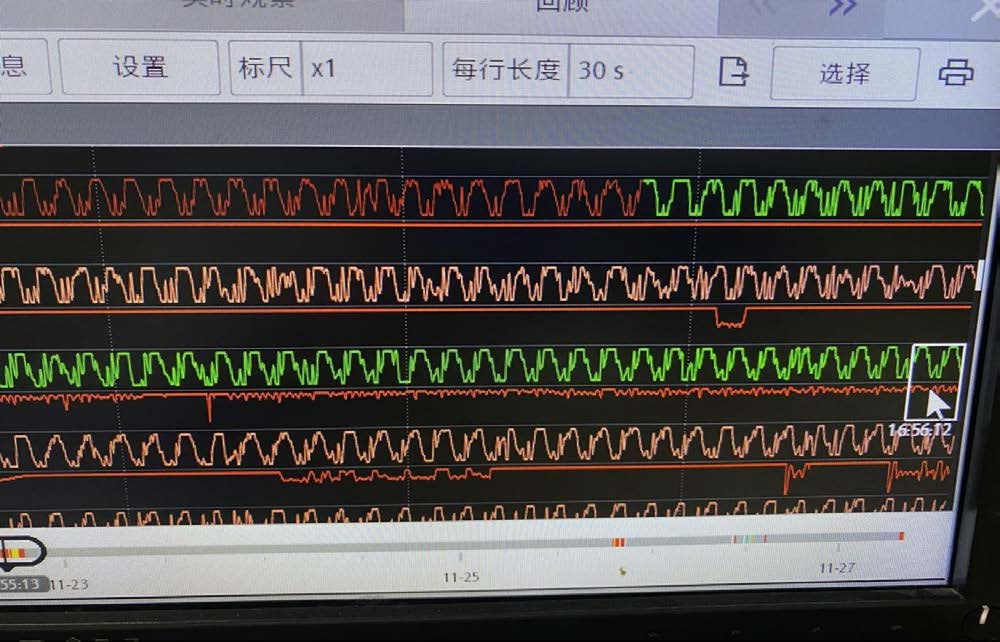
Arterial blood pressure waveform at 16:56 with a very small amplitude.

This invasive blood pressure monitoring technique proved essential in the treatment of cardiac arrest, as it allowed for the observation of arterial blood pressure waveforms, which were less affected by the chest compression in mechanical CPR. Subsequent arterial blood pressure waveforms at 16:56 showed persistent low amplitudes (see Fig. 2).

**Figure 3.**
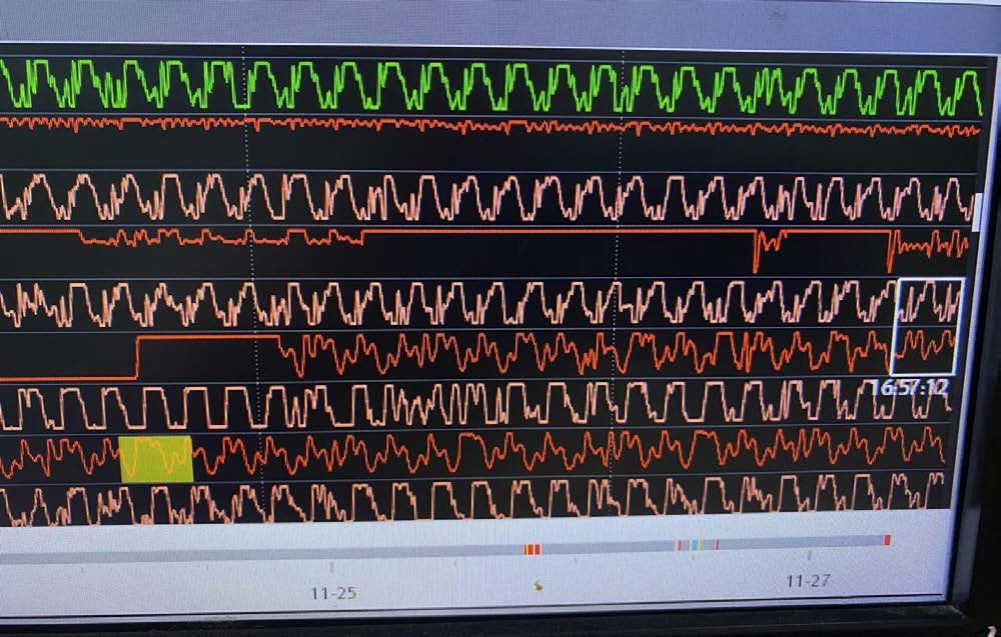
Arterial blood pressure waveform at 16:57 with a sudden increase in amplitude, suggesting the onset of ROSC.

At 16:57, a significant change such as a sudden increase in the amplitude of the arterial pressure waveform was noted, as shown in Figure 3, suggesting the possibility of ROSC.

**Figure 4.**
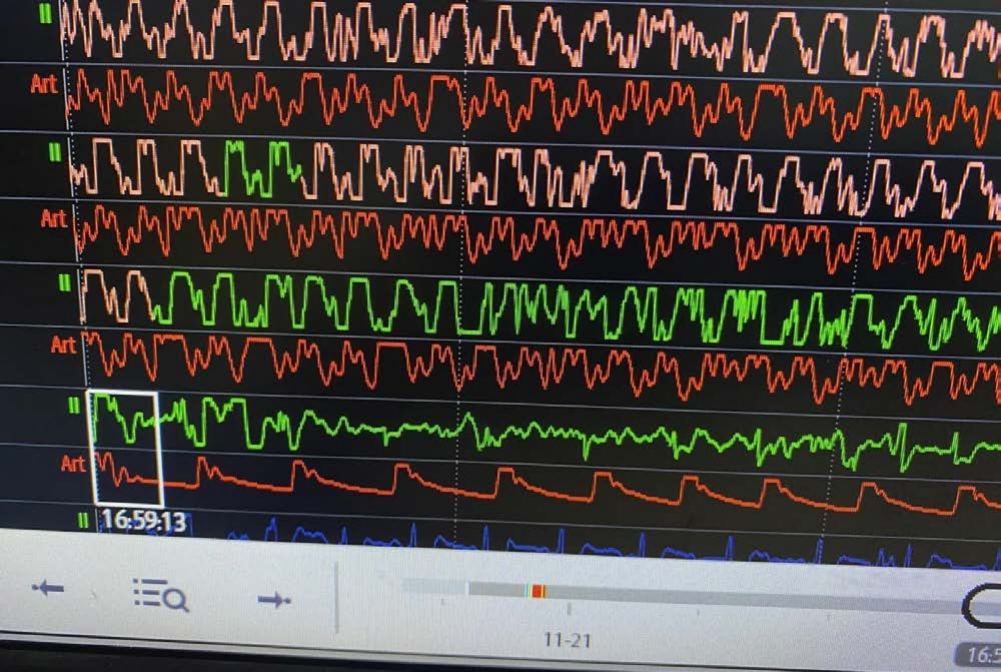
Comparison of arterial blood pressure waveform at 16:59 indicating ROSC with the waveform observed when chest compressions were stopped.

This hypothesis was confirmed at 16:59, when the invasive arterial blood pressure monitoring showed a significant increase in arterial blood pressure, indicating ROSC, which appeared prior to manual confirmation. This pivotal moment is displayed in Figure 4, the arterial blood pressure waveform analysis indicated that ROSC was successful when the chest compression was stopped. The mechanical CPR was discontinued, and the blood pressure was stabilized, thus this patient was transferred to the intensive care unit for further treatment.

Laboratory tests revealed leukocytosis and fluctuating platelet count, which may be due to acute stress response or infection, ongoing platelet consumption or an acute phase reaction induced by disseminated intravascular coagulation, and subsequent increased platelet production in the bone marrow. The coagulation parameters were deranged due to an acute medical condition. Based on the evolution of cardiac biomarkers such as increased high-sensitivity troponin I, creatine kinase-MB, and myoglobin levels; it was presumed that the acute myocardial infarction was a precipitating event for the cardiac arrest (see Table 1 for details).

**Table 1:** Overview of laboratory test results.

Date Time	WBC (×10^9^/L)	RBC (×10^12^/L)	Platelets (×10^9^/L)	PT (s)	aPTT (s)	Antithrombin III (%)	FDP (mg/L)	D-dimer (mg/L)	High-sensitivity troponin I (ng/mL)	CK-MB (ng/mL)	Myoglobin (ng/mL)
November 22 16:40	6.50	3.70	86	18.9	73.6	46.3	100.10	55.80	0.264	19.7	>1200
November 22 18:45	15.79	4.29	106	17.8	55.2	55.3	309	136.00	16.538	-	-
November 23	14.94	3.70	84	32.3	50.4	46.5	98.09	49.60	116.131	-	-

This table shows the laboratory test results of this patient at 3 different time points: immediately after the cardiac arrest, at 2 hours after cardiac arrest, and in the following day. It details the evolution of white and red blood cell counts, platelet levels, coagulation times (PT and aPTT), antithrombin III percentage, fibrin degradation products (FDP), D-dimer levels, and cardiac biomarkers such as hsTnI and CK-MB. These data indicate an acute stress response, potentially ongoing consumption coagulopathy, and significant myocardial injury, which align with the clinical diagnosis of an acute myocardial infarction leading to cardiac arrest.

aPTT = activated partial thromboplastin time, CK-MB = creatine kinase-MB, FDP = fibrin degradation products, hsTnI = high-sensitivity troponin I, mmol/L = millimoles per liter, ng/mL = nanograms per milliliter, PT = prothrombin time, RBC = red blood cells, s = seconds, WBC = white blood cells.

The patient’s treatment included manual CPR, persistent mechanical CPR, advanced airway management, immediate treatment with adrenaline, correction of metabolic acidosis, and radial artery cannulation for invasive monitoring, which was critical not only for guiding fluid resuscitation but also for identifying ROSC.

Although the ROSC in this case was detected by the monitor when the mechanical CPR was stopped, the later review and analysis of the invasive arterial blood pressure waveforms actually indicated that ROSC occurred in this patient prior to our manual examination. This approach underscores the utility of invasive arterial blood pressure monitoring during mechanical CPR as an advanced technique in the treatment of cardiac arrest.

## 3. Discussion

This case report shows the use of the invasive arterial blood pressure monitoring during mechanical CPR and its pivotal role in the early detection of ROSC in an 85-year-old male patient who experienced an OHCA. The discussion delves into the interpretation of clinical findings, comparison with existing literature, and the broader clinical implications.

The notable change in arterial blood pressure waveform and blood pressure detected via radial artery cannulation can provide a real-time indication of ROSC. This immediate feedback is critical, as the early detection of ROSC can minimize the duration of CPR, thereby reducing the incidence of iatrogenic injuries and improving neurological outcomes.^[4]^ In this case, invasive arterial blood pressure monitoring allowed for a more timely and accurate detection of ROSC compared to traditional manual examination, in which detection of ROSC can be affected by the mechanical CPR.^[5]^

A study suggests that mechanical CPR devices can offer a superior consistency in compression depth and rate, potentially leading to improved outcomes compared with manual CPR.^[6]^ However, the integration of invasive arterial blood pressure monitoring in this context is not widely reported. Our findings align with those of Onishi et al,^[7]^ who reported the utility of invasive blood pressure monitoring in detecting ROSC during resuscitation efforts.

The benefits of invasive arterial blood pressure monitoring extend beyond the early detection of ROSC. Continuous arterial blood pressure measurements can provide guidance for resuscitation efforts, including the titration of vasopressors and the management of fluid therapy. Moreover, it allows healthcare providers to focus on other critical aspects of patient management, such as diagnosis and treatment of the underlying cause of cardiac arrest.^[2]^

Despite the advantages presented, the use of invasive arterial blood pressure monitoring must be carefully weighed against the potential risks, such as vascular injury, infection, and the need for skilled personnel to perform the cannulation.^[8]^ Nevertheless, in critical care settings where such expertise is available, the benefits of invasive arterial blood pressure monitoring appear to outweigh the risks, especially in complex cases of OHCA.

This report underscores the need for further research into the adoption of invasive blood pressure monitoring during mechanical CPR. Future studies can explore its effects on survival rate, neurological outcomes, and cost-effectiveness. Additionally, future studies can assess the feasibility of incorporating invasive blood pressure monitoring into advanced life support protocols across various healthcare systems.

## 4. Conclusion

This case underscores the effect of radial artery cannulation for invasive blood pressure monitoring in mechanical CPR. The radial artery cannulation can facilitate an early and accurate detection of ROSC, which is crucial for the appropriate duration and cessation of mechanical CPR and the transition to post-resuscitation care. Integrating such monitoring techniques in the treatment of OHCA can significantly improve patient outcomes by ensuring timely interventions and reducing the duration of cardiac arrest, thereby potentially enhancing survival and neurological outcomes.

## Author contributions

**Data curation:** Li Jin.

**Investigation:** Jianru Xu.

**Writing – original draft:** Li Jin.

**Writing – review & editing:** Hanzhen Ji.
